# Dual-phase FDG PET/CT for predicting prognosis in operable breast cancer

**DOI:** 10.1016/j.breast.2022.07.008

**Published:** 2022-07-19

**Authors:** Haruka Ikejiri, Shinsuke Sasada, Akiko Emi, Norio Masumoto, Takayuki Kadoya, Morihito Okada

**Affiliations:** Department of Surgical Oncology, Research Institute for Radiation Biology and Medicine, Hiroshima University, Hiroshima, Japan

**Keywords:** Breast cancer, PET, FDG, Dual-phase, Prognosis

## Abstract

**Purpose:**

We aimed to investigate the role of dual-phase FDG PET/CT in predicting the prognosis of patients with operable breast cancer.

**Methods:**

We retrospectively reviewed the data of 998 patients who underwent radical treatment for breast cancer. Before treatment, PET/CT scans were performed 1 and 2 h after FDG administration. The maximum standardized uptake value (SUVmax) at both time points (SUVmax1 and SUVmax2) in the primary tumor and the retention index (RI) were calculated. PET recurrence risk (PET-RR) was determined based on the SUVmax1 and RI, and disease-free survival (DFS) and overall survival (OS) were evaluated according to the metabolic parameters. Propensity score matching was performed to adjust for biological characteristics.

**Results:**

The cut-off values for SUVmax1 and RI were 3 and 5%, respectively. The 5-year DFS was 94.9% and 86.1% (*P* < 0.001), and the 5-year OS was 97.6% and 92.7% (*P* < 0.001) in the low and high PET-RR groups, respectively. In multivariate analysis, high T status, nodal metastasis, the triple-negative subtype, and high PET-RR were independent factors of poor DFS. Propensity score matching revealed similar findings (5-year DFS 91.8% vs. 88.6%, *P* = 0.041 and 5-year OS 97.1% vs. 94.2%, *P* = 0.240, respectively).

**Conclusion:**

The combined parameters of SUVmax1 and RI on dual-phase FDG PET/CT were useful for predicting prognosis of patients with breast cancer. Patients with a high SUVmax1 and a negative time course of FDG uptake had a favorable prognosis.

## Highlights

• SUVmax and retention index on FDG PET/CT are related with breast cancer prognosis.
• Breast cancer with high SUVmax but no increase over time has a favorable prognosis.
• Dual-phase FDG PET/CT is useful to predict the prognosis in operable breast cancer.

## Abbreviations

DFS: disease-free survival
ER: estrogen receptor
FDG PET/CT: fluorodeoxyglucose positron emission tomography/computed tomography
HER2: human epidermal growth factor receptor 2
OS: overall survival
PET-RR: positron emission tomography-recurrence risk
PgR: progesterone receptor
RI: retention index
ROC: receiver operating characteristic

## 1 Introduction

Breast cancer is the most prevalent cancer in adult women worldwide and has a favorable prognosis, with a 5-year survival rate of 90% [1]. Although adjuvant systemic therapy is decided based on molecular subtype, risk categories are defined using age, tumor size, grade, extensive peritumoral vascular invasion, hormonal receptors, human epidermal growth factor receptor 2 (HER2)-positivity, and nodal metastasis to identify risk of recurrence [2]. Patients with features that increase the risk of recurrence are indicated for chemotherapy. Recently, multi-gene assays have been developed to identify such patients [3,4].

Fluorodeoxyglucose positron emission tomography/computed tomography (FDG PET/CT) is a molecular imaging technique that focuses on glucose metabolism and detects distant metastases with high sensitivity in patients with breast cancer [5]. In addition, the maximum standardized uptake value (SUVmax) on FDG PET/CT predicts the malignant grade and prognosis of patients with operable breast cancer [6,7]. FDG uptake in malignant tumors increases over time, in contrast to that of benign tumors; thus, delayed-phase PET scans identify high-grade tumors [8]. Furthermore, the retention index (RI), or the rate of change of the SUVmax on dual-phase FDG PET/CT is related to malignant features in breast cancer and identifies nodal metastases [9,10]. Precise prognostic evaluation is important in order to determine the appropriateness of treatment escalation or de-escalation. In addition to tumor biology, evaluation of tumor metabolism might lead to a more accurate prediction of the prognosis. However, the impact of an additional delayed-phase PET scan on prognostic assessment has not been adequately evaluated.

We hypothesized that dual-phase FDG PET/CT would more accurately estimate the prognosis of patients with operable breast cancer than single-phase examination. One retrospective study has reported that the early-phase SUVmax and rate of SUVmax change identify patients with a worse prognosis [11]. However, the report had the limitation of an insufficient follow-up period. Therefore, we evaluated the ability of dual-phase FDG PET/CT to predict prognosis of patients with breast cancer using a large cohort with more than five years of follow-up after surgery.

## 2 Materials and methods

### 2.1 Patients

Consecutive patients with operable stage 0–III breast cancer who underwent pre-treatment dual-phase FDG PET/CT and radical treatment between April 2006 and March 2016 were retrospectively reviewed. FDG PET/CT was performed for preoperative staging on all patients who consented to the procedure. Tumor staging was based on the anatomic stage groups of the 8th edition of the American Joint Committee on Cancer [12]. Postoperative surveillance was performed by physical examination every three to six months and mammography was conducted once a year for five years. Subsequently, annual physical examination and mammography were conducted for up to 10 years after surgery. When recurrence was suspected, diagnosis was determined using CT, FDG PET/CT, or tissue biopsy, if possible.

This study was approved by the institutional review board of Hiroshima University. All procedures performed on human participants were conducted in accordance with the ethical standards of the Institutional Research Committee and the principles of the 1964 Declaration of Helsinki and its later amendments or comparable ethical standards. For this retrospective study, formal consent was not required.

### 2.2 FDG PET/CT imaging

All FDG PET/CT examinations were performed at the same facility using an integrated Discovery.

ST16 PET/CT scanner (GE Healthcare). Patients fasted for at least 4 h before imaging. Dual time-point scans were performed 1 and 2 h after intravenous administration of 3–3.7 MBq/kg FDG. Low-dose non-enhanced CT images (3–4 mm slices) were acquired for attenuation correction and localization of lesions identified on PET images. Immediately after CT examination, the identical axial field of view (154 mm) was scanned using PET for 2–3 min per table position depending on the patient's condition and scanner performance. The acquired data were reconstructed as 128 × 128 matrix images (pixel size, 4.7 × 3.25 mm) using Fourier rebinning and ordered subset expectation maximization algorithms. PET and CT examinations were performed with the patient performing normal tidal breathing in the supine position. Regions of interest were set to include the entire intramammary abnormal uptake on attenuation-corrected FDG PET images. The primary breast tumor and the SUVmax was quantified using a Xeleris workstation (GE Healthcare). Semi-quantitative SUVmax parameters from the first and second scans were defined as SUVmax1 and SUVmax2, respectively. The RI was calculated using the following equation:

$$RI=\frac{SUVmax2-SUVmax1}{SUVmax1}\;\times \;100\;\left(\%\right)$$

### 2.3 Pathological assessment

Histological assessment was performed using surgical or pre-treatment biopsy specimens. The histology, nuclear grade, and presence of nodal metastasis were determined using hematoxylin and eosin-stained tumor slices. Estrogen receptor (ER), progesterone receptor (PgR), and HER2 levels were assessed using immunohistochemistry (IHC) staining according to the guidelines of the American Society of Clinical Oncology/College of American Pathologists [13,14]. ER was scored as either positive or negative with a 1% cut-off value for nuclear immunostaining. HER2-positivity was defined as IHC 3+ or IHC 2+ and gene amplification using fluorescence in situ hybridization. The molecular subtypes of breast cancer were classified as luminal (ER-and/or PgR-positive and HER2-negative), HER2-positive (HER2-positive regardless of ER and PgR-positivity), or triple-negative (ER-, PgR-, and HER2-negative). The luminal breast cancer types were classified as luminal A-like (Ki-67 labelling index <20% and nuclear grade 1–2) and luminal B-like (Ki-67 labelling index ≥20% and/or nuclear grade 3) [15].

### 2.4 Statistical analysis

Data are presented as numbers and percentages unless otherwise stated. The chi-squared test was used to compare the frequencies of categorical variables. Continuous variables were compared using the Kruskal–Wallis test. The cut-off values for SUVmax and RI were determined using the receiver operating characteristic (ROC) curve. Disease-free survival (DFS) and overall survival (OS) were analyzed using the Kaplan–Meier method with the log-rank test. Prognostic factors were assessed by univariate and multivariate analyses using the Cox proportional hazards model. Propensity score matching (1:1) including T status, N status, nuclear grade, and subtype, was performed using a calliper width of 0.2 to control for confounding due to differences in the prognostic characteristics between the PET parameter groups. Statistical significance was set at *P* < 0.05. Statistical analyses were performed using EZR 1.54 (Saitama Medical Center, Jichi Medical University, Saitama, Japan), a graphical user interface for R version 4.0.3 (The R Foundation for Statistical Computing, Vienna, Austria) [16].

## 3 Results

The clinicopathological characteristics of the 998 patients are shown in Supplementary Table S1. Regarding subtype, 338 (33.9%) luminal A-like, 412 (41.3%) luminal B-like, 153 (15.3%) HER2-positive, and 93 (9.3%) triple-negative cases were observed. Neoadjuvant chemotherapy was administered to 177 (17.7%) patients. The median SUVmax1, SUVmax2, and RI values were 2.3, 2.4, and 4.2%, respectively. According to the ROC curves, the optimal cut-off values for the SUVmax1 and RI were 3 and 5%, respectively. The patients were divided into four groups according to high (>3) and low SUVmax1 (≤3), and high (>5%) and low RI (≤5%). Additionally, the four groups were classified as high (high SUVmax1/high RI) and low (low SUVmax1/low RI, low SUVmax1/high RI, or high SUVmax1/low RI) PET-recurrence risk (PET-RR) based on the prognosis. Table 1 shows the patients’ characteristics according to PET-RR.

**Table 1:** Patient characteristics according to PET-recurrence risk.

	Low PET-RR	High PET-RR	*P*
	(n = 702)	(n = 296)	
Age (y), median (range)	59 (29–91)	61 (28–90)	0.678
Histology			<0.001
Ductal carcinoma in situ	120 (17.1)	5 (1.7)	
Infiltrating duct carcinoma, NOS	505 (71.9)	273 (92.2)	
Lobular carcinoma, NOS	19 (2.7)	4 (1.4)	
Others	58 (8.3)	14 (4.7)	
T status			<0.001
Tis	120 (17.1)	5 (1.7)	
T1	425 (60.5)	103 (34.8)	
T2	140 (19.9)	151 (51.0)	
T3	11 (1.6)	18 (6.1)	
T4	6 (0.9)	19 (6.4)	
N status			<0.001
N0	549 (78.2)	144 (48.6)	
N1	121 (17.2)	105 (35.5)	
N2	23 (3.3)	30 (10.1)	
N3	9 (1.3)	17 (5.7)	
Stage			<0.001
0	120 (17.1)	5 (1.7)	
I	353 (50.3)	70 (23.6)	
II	188 (26.8)	153 (51.7)	
III	41 (5.8)	68 (23.0)	
Nuclear grade			<0.001
1	143 (20.4)	14 (4.7)	
2	308 (43.9)	100 (33.9)	
3	250 (35.7)	181 (61.4)	
Unknown			
Subtype			<0.001
Luminal A-like	287 (40.9)	51 (17.2)	
Luminal B-like	273 (38.9)	139 (47.0)	
HER2-positive	96 (13.7)	57 (19.3)	
Triple-negative	44 (6.3)	49 (16.6)	
Unknown	2 (0.3)	0 (0)	

HER2, human epidermal growth factor receptor 2; IQR, interquartile range; NOS, not otherwise specified; PET-RR, positron emission tomography-recurrence risk; RI, retention index; SUVmax, maximum standardized uptake value.

**Fig. 1:**
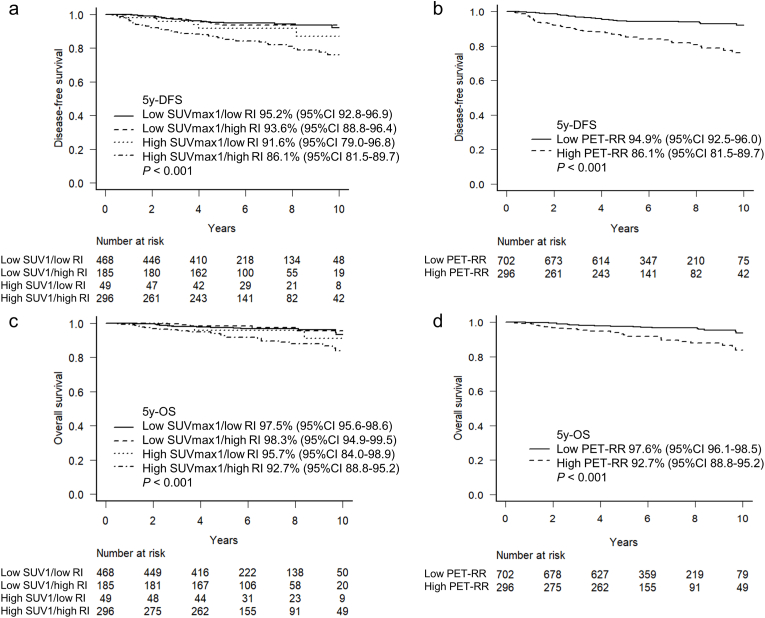
DFS and OS curves according to metabolic parameters. DFS curves according to SUVmax1/RI (a) and PET-RR (b). OS curves according to SUVmax1/RI (c) and PET-RR (d). DFS, disease-free survival; OS, overall survival; PET-RR, positron emission tomography-recurrence risk; RI, retention index; SUVmax, maximum standardized uptake value.

**Supplementary Fig. S1:**
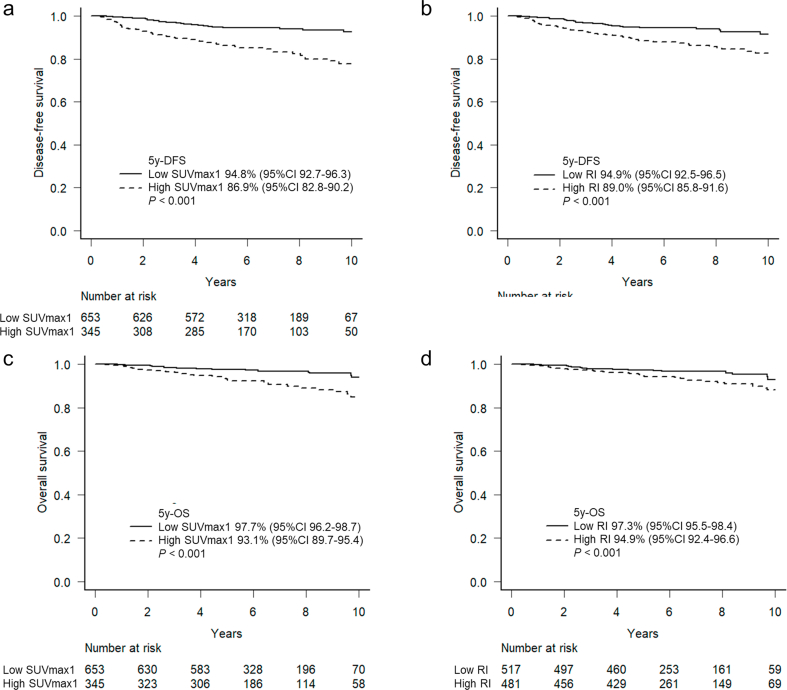
Disease-free survival and overall curves according to SUVmax1 and RI. RI, retention index; SUVmax, maximum standardized uptake value.

The median follow-up period was six years. Additionally, the DFS differed significantly according to SUVmax1 and RI, and low SUVmax1 and RI were associated with favorable DFS and OS (Supplementary Fig. S1). The five-year DFS was 95.2% in the low SUVmax1/low RI group, 93.6% in the low SUVmax1/high RI group, 91.6% in the high SUVmax1/low RI group, and 86.1% in the high SUVmax1/high RI group (*P* < 0.001, Fig. 1a). The five-year DFS rate of the low PET-RR group was significantly higher than that of the high PET-RR group (94.9% vs. 86.1%, *P* < 0.001, Fig. 1b). In a multivariate Cox proportional hazards analysis, high T status, presence of nodal metastasis, the triple-negative subtype, and high PET-RR were independent factors of poor DFS (Table 2). Similar trends were observed in the OS curves. The five-year OS rate was 97.5% in the low SUVmax1/low RI group, 98.3% in the low SUVmax1/high RI group, 95.2% in the high SUVmax1/low RI group, 92.7% in the high SUVmax1/high RI group, and 97.6% in the low PET-RR group (*P* < 0.001, Fig. 1c and d).

**Table 2:** Cox proportional hazards analysis for predicting disease-free survival.

Factors	Univariate analysis		Multivariate analysis	
	HR (95% CI)	*P*	HR (95% CI)	*P*
Age <50y	0.84 (0.53–1.35)	0.479	0.73 (0.45–1.18)	0.195
T2–4	2.89 (1.92–4.37)	<0.001	1.85 (1.16–2.95)	0.010
Nodal metastasis	2.61 (1.74–3.93)	<0.001	1.73 (1.11–2.70)	0.016
Nuclear grade 3	1.31 (0.87–1.97)	0.195	0.91 (0.54–1.54)	0.731
Subtype				
Luminal A-like	Ref		Ref	
Luminal B-like	1.25 (0.75–2.07)	0.397	0.97 (0.52–1.79)	0.918
HER2-positive	1.05 (0.53–2.09)	0.893	0.77 (0.34–1.72)	0.522
Triple-negative	2.99 (1.65–5.43)	<0.001	2.11 (1.03–4.29)	0.040
High PET-RR	3.05 (2.02–4.58)	<0.001	2.05 (1.30–3.23)	0.002

CI, confidence interval; HER2, human epidermal growth factor receptor 2; HR, hazard ratio; PET-RR, positron emission tomography-recurrence risk.

**Fig. 2:**
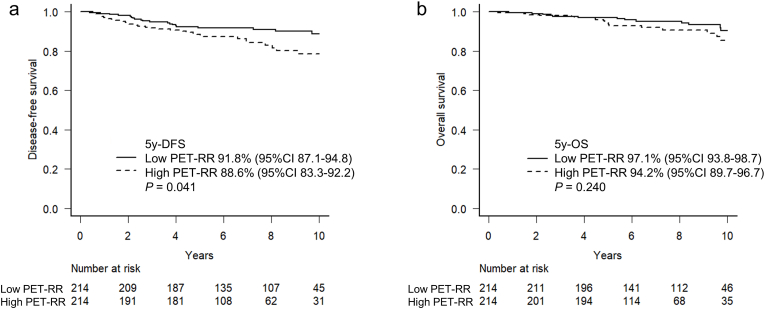
DFS (a) and OS (b) curves according to PET-RR with propensity score matching. DFS, disease-free survival; OS, overall survival; PET-RR, positron emission tomography-recurrence risk.

**Fig. 3:**
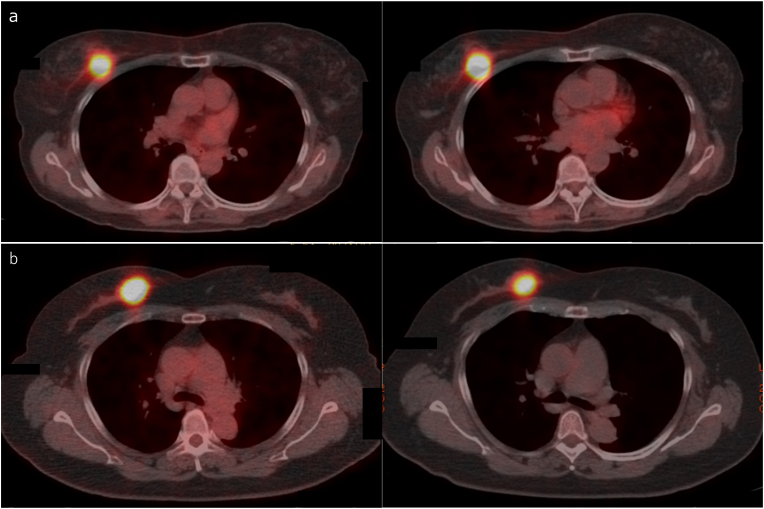
Representative images of FDG PET/CT at early (left) and delayed (right) phases. (a) A 67-year-old woman with infiltrating duct carcinoma not otherwise specified, T2N0M0 stage IIA, luminal B-like subtype, nuclear grade 2, SUVmax1 10.2, SUVmax2 15.6, RI 52.9% in right breast. The patient experienced recurrence of bone metastasis 29 months after surgery and died 60 months after surgery. (b) A 63-year-old woman with infiltrating duct carcinoma not otherwise specified, T2N0M0 stage IIA, luminal B-like subtype, nuclear grade 2, SUVmax1 16.8, SUVmax2 17.2, RI 2.4% in right breast. The patient remained relapse-free 98 months after surgery. RI, retention index; SUVmax, maximum standardized uptake value.

Among 214 pairs from propensity score matching for high and low PET-RR, the five-year DFS of the low PET-RR group was significantly higher than that of the high PET-RR group (91.8% vs. 88.6%, *P* = 0.041, Table 3 and Fig. 2a). However, no significant difference in the OS between the two groups was observed (97.1% vs. 94.2%, *P* = 0.240, Fig. 2b). Fig. 3 shows representative images of patients with similar pathological features from the high and low PET-RR groups. A patient with high PET-RR experienced recurrence of bone metastases 29 months after surgery (Fig. 3a), while a patient with low PET-RR was free of recurrence for 98 months after surgery (Fig. 3b).

**Table 3:** Characteristics of propensity score-matched patients according to PET-recurrence risk.

	Low PET-RR	High PET-RR	*P*
	(n = 214)	(n = 214)	
Age (y), median (range)	59 (29–88)	61 (28–90)	0.620
Histology			0.392
Ductal carcinoma in situ	5 (2.3)	5 (2.3)	
Infiltrating duct carcinoma, NOS	186 (86.9)	196 (91.6)	
Lobular carcinoma, NOS	6 (2.8)	3 (1.4)	
Others	17 (7.9)	10 (4.7)	
T status			1
Tis	5 (2.3)	5 (2.3)	
T1	97 (45.3)	97 (45.3)	
T2	104 (48.6)	104 (48.6)	
T3	5 (2.3)	5 (2.3)	
T4	3 (1.4)	3 (1.4)	
N status			1
N0	125 (58.4)	125 (58.4)	
N1	71 (33.2)	71 (33.2)	
N2	13 (6.1)	13 (6.1)	
N3	5 (2.3)	5 (2.3)	
Stage			0.997
0	5 (2.3)	5 (2.3)	
I	68 (31.8)	66 (30.8)	
II	117 (54.7)	119 (55.6)	
III	24 (11.2)	24 (11.2)	
Nuclear grade			1
1	10 (4.7)	10 (4.7)	
2	78 (36.4)	78 (36.4)	
3	126 (58.9)	126 (58.9)	
Subtype			1
Luminal A-like	42 (19.6)	42 (19.6)	
Luminal B-like	116 (54.2)	116 (54.2)	
HER2-positive	36 (16.8)	36 (16.8)	
Triple-negative	20 (9.3)	20 (9.3)	

HER2, human epidermal growth factor receptor 2; NOS, not otherwise specified; PET-RR, positron emission tomography-recurrence risk.

## 4 Discussion

This study demonstrated the ability of dual-phase FDG PET/CT to predict the prognosis of patients with operable breast cancer. The combined parameters of SUVmax1 and RI were more useful for prognosis than the individual parameters.

Glucose consumption is correlated with the proliferation of cancer cells, and high FDG accumulation is indicative of aggressive tumors [17,18]. Previously, we reported that a high SUVmax1 (>3.0) is an independent factor of poor prognosis [6]. A recent meta-analysis also demonstrated that the pre-treatment early-phase SUVmax was a significant prognostic factor, especially for patients with the luminal subtype of breast cancer. That study suggested more aggressive treatment for patients with a high SUVmax [19]. However, the SUVmax cut-off values varied and were not uniform (range: 2.9–11.1) due to differences in imaging devices, stages, and subtypes of target cases.

The SUVs of malignant tumors can increase for up to 4 h, unlike those of benign tumors [8,20]. Some studies have reported that RI is related to the biological characteristics of breast cancer [9,21,22]. RI has also been reported to be correlated with prognosis in patients with lung cancer, cholangiocarcinoma, and lymphoma [[23], [24], [25]]. However, only one study has reported a relationship between RI and prognosis in patients with breast cancer [11]. The study suggested that the combination of SUVmax1 and RI was more useful for predicting prognosis than SUVmax1 alone. Although the median follow-up period was 4.9 years, patients were eligible after only 1 year of observation, and 5- and 10-year survival rates were immature. Moreover, biological characteristics were not considered in the analysis using the Kaplan–Meier method. Nevertheless, our study boasts the strengths of a large cohort, a sufficient follow-up period longer than five years, and the use of propensity score matching. We observed that patients with a high SUVmax1 and a low RI had similar DFS and OS curves to those in the low SUVmax1 group. Therefore, patients with a high SUVmax1/low RI, as well as those with a low SUVmax1 were classified as low PET-RR. Combining SUVmax1 and RI parameters stratified DFS, even after adjusting for biological characteristics. Finally, our study suggests that approximately 14% of cases with a high SUVmax1 can avoid overtreatment.

The significance of RI is limited to cases with significant FDG uptake in the early phase because changes in the SUVmax are influenced by the physiological accumulation of FDG in normal mammary glands when SUVmax1 is low. In this study, the median RI of normal mammary glands was 0% (interquartile range: −12.5%–6.25%), and a cut-off value of 5% reflected the physiological variability in FDG uptake.

In this study, 17.7% of the patients received neoadjuvant chemotherapy. Pathological response after neoadjuvant chemotherapy predicts the risk of recurrence in early breast cancer. Although pathological complete response rate varies by subtype, many patients have residual disease following neoadjuvant chemotherapy, regardless of the subtype [26]. It remains unclear whether SUV and RI values are predictors of the response to treatment or prognostic factors after neoadjuvant chemotherapy, and further investigations are required.

This study was limited by its retrospective nature and single-institutional design. The SUV value is a semi-quantitative parameter and there may be inter-institutional variability. In a previous multicenter study, the SUV value was adjusted using a phantom [6]. Thus, a prospective, multicenter study is required to validate the results of this study. In addition, no comparison was made between the prognostic value of dual-phase FDG PET/CT and multi-gene assays. Although no data exist to justify recommending routine FDG PET/CT testing for patients with early-stage breast cancer, further investigations are required to determine whether metabolic parameters provide useful prognostic information in addition to multi-gene assays.

## 5 Conclusion

This study investigated the ability of metabolic parameters of dual-phase FDG PET/CT to predict prognosis in patients with operable breast cancer. Dual-phase FDG PET/CT was useful for predicting prognosis. Finally, patients with breast cancer with a high SUVmax1 and a negative time course of FDG uptake experienced a favorable prognosis.

## Funding

None.

## Declaration of competing interest

The authors declare that they have no conflict of interest.
