# Supplementary material for: Dual-phase FDG PET/CT for predicting prognosis in operable breast cancer
Source: Breast. 2022 Jul 19;65:98–103. doi: 10.1016/j.breast.2022.07.008 (PMC9344020; doi:10.1016/j.breast.2022.07.008)
Supplement: Multimedia component 1 [file mmc1.docx]

Supplementary Table S1. Patient characteristics according to SUVmax and RI.

|  | Total | SUVmax ≤ 3/RI ≤ 5% | SUVmax ≤ 3/RI > 5% | SUVmax > 3/RI ≤ 5% | SUVmax > 3/RI > 5% |
| --- | --- | --- | --- | --- | --- |
|  | (n = 998) | (n = 468) | (n = 185) | (n = 49) | (n = 296) |
| Age (y), median (range) | 59 (28–91) | 59 (29–91) | 60 (33–89) | 57 (37–87) | 61 (28–90) |
| Histology |  |  |  |  |  |
| Ductal carcinoma in situ | 125 (12.5) | 101 (21.6) | 19 (10.3) | 0 (0) | 5 (1.7) |
| Infiltrating duct carcinoma, NOS | 778 (78.0) | 311 (66.5) | 151 (81.6) | 43 (87.8) | 273 (92.2) |
| Lobular carcinoma, NOS | 23 (2.3) | 18 (3.8) | 1 (0.5) | 0 (0) | 4 (1.4) |
| Others | 72 (7.2) | 38 (8.1) | 14 (7.6) | 6 (12.2) | 14 (4.7) |
| T status |  |  |  |  |  |
| Tis | 125 (12.5) | 101 (21.6) | 19 (10.3) | 0 (0) | 5 (1.7) |
| T1 | 528 (52.9) | 287 (61.3) | 120 (64.9) | 18 (36.7) | 103 (34.8) |
| T2 | 291 (29.2) | 70 (15.0) | 42 (22.7) | 28 (57.1) | 151 (51.0) |
| T3 | 29 (2.9) | 6 (1.3) | 3 (1.6) | 2 (4.1) | 18 (6.1) |
| T4 | 25 (2.5) | 4 (0.9) | 1 (0.5) | 1 (2.0) | 19 (6.4) |
| N status |  |  |  |  |  |
| N0 | 693 (69.4) | 384 (82.1) | 138 (74.6) | 27 (55.1) | 144 (48.6) |
| N1 | 226 (22.7) | 67 (14.3) | 40 (21.6) | 14 (28.6) | 105 (35.5) |
| N2 | 53 (5.3) | 13 (2.8) | 5 (2.7) | 5 (10.2) | 30 (10.1) |
| N3 | 26 (2.6) | 4 (0.9) | 2 (1.1) | 3 (6.1) | 17 (5.7) |
| Stage |  |  |  |  |  |
| 0 | 125 (12.5) | 101 (21.6) | 19 (10.3) | 0 (0) | 5 (1.7) |
| I | 423 (42.4) | 245 (52.4) | 95 (51.4) | 13 (26.5) | 70 (23.6) |
| II | 341 (34.2) | 101 (21.6) | 60 (32.4) | 27 (55.1) | 153 (51.7) |
| III | 109 (10.9) | 21 (4.5) | 11 (5.9) | 9 (18.4) | 68 (23.0) |
| Nuclear grade |  |  |  |  |  |
| 1 | 157 (15.7) | 110 (23.5) | 29 (15.8) | 4 (8.2) | 14 (4.7) |
| 2 | 408 (40.9) | 203 (43.4) | 84 (45.7) | 21 (42.9) | 100 (33.9) |
| 3 | 431 (43.2) | 155 (33.1) | 71 (38.6) | 24 (49.0) | 181 (61.4) |
| Unknown | 2 (0.2) | 0 (0) | 0 (0) | 0 (0) | 0 (0) |
| Subtype |  |  |  |  |  |
| Luminal A-like | 338 (33.9) | 214 (45.7) | 63 (34.1) | 10 (20.4) | 51 (17.2) |
| Luminal B-like | 412 (41.3) | 167 (35.7) | 85 (45.9) | 21 (42.9) | 139 (47.0) |
| HER2-positive | 153 (15.3) | 57 (12.2) | 26 (14.1) | 13 (26.5) | 57 (19.3) |
| Triple-negative | 93 (9.3) | 28 (6.0) | 11 (5.9) | 5 (10.2) | 49 (16.6) |
| Unknown | 2 (0.2) | 4 (0.4) | 0 (0) | 0 (0) | 0 (0) |
| SUVmax1, median (IQR) | 2.3 (1.6–4.2) | 1.7 (1.2–2.1) | 2.0 (1.6–2.7) | 4.4 (3.8–5.4) | 5.6 (4.2–8.0) |
| SUVmax2, median (IQR) | 2.4 (1.4–4.7) | 1.5 (1.0–2.1) | 2.3 (1.7–3.0) | 4.4 (3.8–5.2) | 6.5 (4.9–9.7) |
| RI (%), median (IQR) | 4.2 (-7.7–15.6) | -8.3 (-16.7–0.0) | 12.5 (7.7–17.7) | 0.0 (₋3.0–2.6) | 18.8 (12.9–25.3) |

HER2, human epidermal growth factor receptor 2; IQR, interquartile range; NOS, not otherwise specified; RI, retention index; SUVmax, maximum standardized uptake value.
